# The chemical identity of intervessel pit membranes in *Acer* challenges hydrogel control of xylem hydraulic conductivity

**DOI:** 10.1093/aobpla/plw052

**Published:** 2016-08-02

**Authors:** Matthias M. Klepsch, Marco Schmitt, J. Paul Knox, Steven Jansen

**Affiliations:** ^1^Institute for Systematic Botany and Ecology, Ulm University, Albert-Einstein-Allee 11, D-89081 Ulm, Germany; ^2^Centre for Plant Sciences, Faculty of Biological Sciences, University of Leeds, Leeds LS2 9JT, UK

**Keywords:** Acer, glycoproteins, hydraulic conductivity, immunocytochemistry, ionic effect, pectic polysaccharides, pit membrane, vessel

## Abstract

Ion-mediated enhancement of the hydraulic conductivity of xylem tissue (i.e. the ionic effect) has been reported for various angiosperm species. One explanation of the ionic effect is that it is caused by the swelling and shrinking of intervessel pit membranes due to the presence of pectins and/or other cell-wall matrix polymers such as heteroxylans or arabinogalactan–proteins (AGPs) that may contain acidic sugars. Here, we examined the ionic effect for six *Acer* species and their pit membrane chemistry using immunocytochemistry, including antibodies against glycoproteins. Moreover, anatomical features related to the bordered pit morphology and vessel dimensions were investigated using light and electron microscopy. The ionic effect varied from 18 % (± 9) to 32 % (± 13). Epitopes of homogalacturonan (LM18) and xylan (LM11) were not detected in intervessel pit membranes. Negative results were also obtained for glycoproteins (extensin: LM1, JIM20; AGP glycan: LM2), although AGP (JIM13)-related epitopes were detected in parenchyma cells. The mean vessel length was significantly correlated with the magnitude of the ionic effect, unlike other pit or vessel-related characteristics. Our results suggest that intervessel pit membranes of *Acer* are unlikely to contain pectic or other acidic polysaccharides. Therefore, alternative explanations should be tested to clarify the ionic effect.

## Abstract (precis)

Long-distance water transport in wood is well known to be affected by the ionic concentration of xylem sap. Based on immunocytochemistry, we demonstrate that pectic polysaccharides and glycoproteins are not associated with vessel-vessel pit membranes. Therefore, we challenge the hypothesis that the ion-mediated enhancement of hydraulic conductance is caused by a swelling or shrinking of pectins surrounding pit membrane pores. Our findings reinforce the need for an alternative hypothesis besides the pit membrane chemistry to understand this so-called “ionic effect”.

## Introduction

According to the cohesion–tension theory, long-distance water transport in plants occurs through the xylem tissue in a passive way (Askenasy 1895; Dixon and Joly 1895; Jansen & Schenk 2015). The driving force for water uptake is set by the transpiration rate in leaves and is under stomatal control (Damour *et al.* 2010). In angiosperm xylem, individual vessel elements dissolve their primary and secondary cell wall partially to form perforated, multicellular vessels that are specialised for water transport. However, these stacks of vessel elements are of finite length, which means that no individual vessel provides a direct connection from the roots to the canopy of a tree. Instead, water is transported through an interconnected network of vessels, which is enabled by thousands of bordered pits between neighbouring vessel walls (Choat *et al.* 2008).

The micromorphology of bordered pits between adjacent vessel walls and especially the intervessel pit membrane is assumed to play a key role in drought-induced embolism formation (Lens *et al.* 2011; Scholz *et al.* 2013a; Schenk *et al.* 2015) and regulating hydraulic resistance (Sperry *et al.* 2005; Wheeler *et al.* 2005; Choat *et al.* 2006). Several studies reported that xylem hydraulic conductance may depend on the pH, the ionic strength, and ionic identity of the perfused solvents (Zimmermann 1978; van Ieperen 2007; Nardini *et al.* 2012). Our mechanistic understanding of this so-called ‘ionic effect’, however, remains limited. Frequently cited explanations for the ionic effect include the hydrogel hypothesis (Zwieniecki *et al.* 2001) and the electroviscocity hypothesis (van Doorn *et al.* 2011; Santiago *et al.* 2013), which both rely on chemical and physical properties of intervessel pit membranes.

According to the hydrogel hypothesis, the resistance of the water molecules through the porous network of pit membrane microfibrils is affected by a potential swelling or shrinking of pectins, which are a highly heterogeneous class of acidic polysaccharides (Bonner 1946; Caffall and Mohnen 2009; Kastner *et al.* 2012). The backbone of pectins is a linear chain of (1-4)-linked α-d-galactosyluronic residues (homogalacturonan, HG), which can be modified in various ways, most notably by methylesterification to generate acidic residues. Unesterified galactosyluronic residues of pectic HG can interact with cations in the xylem sap and have been suggested to result in a swelling or shrinking of pectins (Kastner *et al.* 2012; Ngouémazong *et al.* 2012). Pectic HG with higher degrees of methylesterification can also form gels at low pH in the presence of saccharides such as sucrose through mechanisms involving hydrophobic interactions and hydrogen bonds (Kastner *et al.* 2012). Other modifications of HG in addition to methyl-esterification, include acetylation of individual monomers, or substitution by xylosyl residues. Introduction of rhamnose to the galacturonic acid-rich backbone can lead to branching with neutral residues (Caffall and Mohnen 2009). If pectins were an integral component of the pit membrane, then its swelling could increase hydraulic resistance by reducing the diameter of the nanoscale pores between the cellulose microfibrils of the pit membrane (Zwieniecki *et al.* 2001).

Previous studies suggest that pectins disappear during the final stages of vessel development by hydrolytic enzymes that remove the non-cellulosic components (O’Brien 1970; Kim and Daniel 2012; Kim and Daniel 2013; Herbette *et al.* 2015). Although about 20 different antibodies have already been applied on bordered pit membranes (Table 1), immunocytochemistry techniques are limited to few species only, especially *Populus* and *Vitis vinifera*. Most studies show that pectic polysaccharides are absent in the actual, fully developed pit membrane, but present in the outermost rim of the pit membrane (i.e. the annulus) and in immature pit membranes of developing vessel elements (Table 1; Wydra and Beri 2007; Plavcová and Hacke 2011; Kim and Daniel 2013; Herbette *et al.* 2015). An exception to this is the report of pectins based on JIM7 in juvenile shoots of *Vitis vinifera* (Sun *et al.* 2011). Moreover, the ionic effect did not decrease in transgenic plants of *Nicotiana tabacum* (PG7 and PG16) with reduced HG content in comparison with wild-type plants with assumingly higher pectin levels (Nardini *et al.* 2007a). If pectin is lacking in intervessel pit membranes, it is possible that other acidic cell-wall matrix polysaccharides/proteoglycans, such as heteroxylans or AGPs, could show a similar swelling and shrinking behaviour as pectins (Li and Pan 2010), although there is also little evidence for their distribution in intervessel pit membranes (Table 1; van Doorn *et al.* 2011). Xyloglucan (LM15) and mannan (LM21) epitopes were found to be absent in mature bordered pit membranes of hybrid poplar and hybrid aspen (Kim and Daniel 2013; Herbette *et al.* 2015), while xyloglucan was found in intervessel pit membranes of juvenile grapevine stems and European aspen (Sun *et al.* 2011; Kim and Daniel 2013).

**Table 1.** Overview of antibodies tested on pits in angiosperm xylem tissue. PM = intervessel pit membrane; Par PM = parenchyma pit membrane; HG = homogalacturonan; RG = rhamnogalacturonan-I; Me = methylesterified; AGP = arabinogalactan-protein: + = strong to weak signals detected; − = negative staining.

Antibody	Epitope	Reference	Species	PM	PM annulus	Parenchyma PM	Comment
1. *Pectic polysaccharides*							
2F4	Non-Me galacturonic acid blocks dimerized by calcium	Herbette *et al*. 2015	*Populus tremula x alba*	*	+	+	* = Only immature pits
JIM5	Partially Me-HG/de-esterified HG	Wydra and Beri 2007	*Solanum lycopersicum (*genotype L390 and L7996)				+ For parenchyma and vessel walls after incubating with *Ralstonia solanacearum* strain To-udk2
		Plavcová and Hacke 2011	*Betula papyrifera, Populus balsamifera, Prunus virginiana*, *Amelanchier alnifolia*	–	+	+	
JIM7	Partially Me-HG	Wydra and Beri 2007	*Solanum lycopersicum (*genotype L390 and L7996)	–	–	–	+ For parenchyma and vessel walls after incubating with *Ralstonia solanacearum* strain To-udk2
		Plavcová and Hacke 2011	*Betula papyrifera, Populus balsamifera, Prunus virginiana*, *Amelanchier alnifolia*	–	+	+	
		Sun *et al*. 2011	*Vitis vinifera* var. Chardonnay and var. Riesling	–	–	–	
LM5	(1→4)-β-galactan side chains of RG	Wydra and Beri 2007	*Solanum lycopersicum* (genotype L390 and L7996)				Parenchyma cell walls
		Herbette *et al*. 2015	*Populus tremula x alba*	–	–		
LM6	(1→5)-α-I-arabinan of RG	Wydra and Beri 2007	*Solanum lycopersicum* (genotype L390 and L7996)	–	–		
		Plavcová and Hacke 2011	*Betula papyrifera, Populus balsamifera, Prunus virginiana*, *Amelanchier alnifolia*	+	+	–	
		Herbette *et al*. 2015	*Populus tremula x alba*	–	–		
LM7	Non-blockwise de-esterification of HG	Wydra and Beri 2007	*Solanum lycopersicum* (genotype L390 and L7996)	–	–		
LM19	Low Me HG epitopes	Kim and Daniel 2013	*Populus tremula × P. tremuloides*, *P. tremula*	+*	+*	+	* = Only immature pits
LM20	High Me HG epitopes	Kim and Daniel 2013	*Populus tremula × P. tremuloides*, *P. tremula*	+*	+*	+	* = Only immature pits
		Herbette *et al*. 2015	*Populus tremula x alba*	+*	+		* = Only immature pits
RU1	RG	Herbette *et al*. 2015	*Populus tremula x alba*	+*	+		* = Only immature pits
2. *Non-cellulosic, non-pectic polysaccharides*							
BMG C6	Galactoglucomannan	Kim and Daniel 2012	*Populus tremula*				Weak signal in ray parenchyma and vessel walls
LM10	Xylan	Kim and Daniel 2012	*Populus tremula*				All cell walls in the xylem
LM11	Xylan	Kim and Daniel 2012	*Populus tremula*				All cell walls in the xylem
LM15	Xylogulcans	Plavcová and Hacke 2011	*Betula papyrifera, Populus balsamifera, Prunus virginiana*, *Amelanchier alnifolia*	+	+	+	
		Kim and Daniel 2013	*Populus tremula × P. tremuloides*, *P. tremula*	+*	+*	+	* = Only immature pits
		Herbette *et al*. 2015	*Populus tremula x alba*	–	–		No staining
CCRC-M1	Fucosylated xyloglucan	Sun et al. 2011	*V. vinifera*	+	+	–	
LM21	Mannan	Kim and Daniel 2012	*Populus tremula*				Weak signal in ray parenchyma and vessel walls
		Herbette *et al*. 2015	*Populus tremula x alba*	–	–		
3. *Plant cell-wall proteogylcans/glycoproteins*							
AX1	arabinoxylans	Herbette *et al*. 2015	*Populus tremula x alba*				Only pit borders
LM2	AGP	Wydra and Beri 2007	*Solanum lycopersicum* (genotype L390 and L7996)				Metaxylem vessel walls
4. *Lignin*							
Anti-S	Non-condensed lignin homosyringyl substructure	Herbette *et al*. 2015	*Populus tremula x alba*	+	+		+ Also for pit borders
Anti-GS	Non-condensed lignin mixed guaiacyl-syringyl structure (75 % syringyl units)	Herbette *et al*. 2015	*Populus tremula x alba*	+	+		+ Also for pit borders
Anti-G	Condensed lignin homoguaiacyl – substructure	Herbette *et al*. 2015	*Populus tremula x alba*	+	+		+ Also for pit borders
Anti**-**protein**-**coupled βGlcY	Protein-coupled βGlcY	Göllner *et al*. 2013	–	–	–	+ for trachery cell walls	

This paper aims to further test the hydrogel hypothesis by investigating the ionic effect and the chemical composition of pit membranes in six closely related *Acer* species. Because most of the earlier evidence indicates that HG-related pectic epitopes (JIM5, JIM7, LM7, LM 19 and LM20) and rhamnogalacturonan (RG)-I-related pectic epitopes (LM5 and LM6) could not be detected in intervessel pit membranes (Table 1), we limited our selection of HG-related antibodies to LM18, which has not been applied to pit membranes as far as we know, while LM11 was chosen as a heteroxylan antibody, and four antibodies (LM1, LM2, JIM13 and JIM20) were selected to test for the presence of glycoproteins, including extensin and AGP glycans. Glycoproteins have not been reported in the actual membrane of bordered pits (Wydra and Beri 2007; Herbette *et al.* 2015), although proteins and AGPs occur in xylem sap and may accumulate in pit membranes of vessel elements and tracheids (Iwai *et al.* 2003; Buhtz *et al.* 2004). Whether or not (glyco)proteins play a role in the ionic effect is unknown (Neumann *et al.* 2010).

In addition, we investigated anatomical features related to pit and vessel dimensions to better understand structural characters associated with the ionic effect. Earlier work suggests that the amount of intervessel pit membrane area per vessel is associated with the magnitude of the ionic effect, both across taxonomically unrelated species and four related species of *Acer* (Jansen *et al.* 2011; Nardini *et al.* 2012). Therefore, we expect that two scenarios could explain the magnitude of the ionic effect in six *Acer* species: (1) the chemical identity and/or the anatomy of intervessel pit membranes, or (2) none of these two, which means that alternative explanations would be required.

## Methods

### Plant material

We collected 1- to 3-year-old branches from five *Acer* species (*Acer campestre*, *A. monspessulanum*, *A. palmatum*, *A. sieboldianum* and *A. tataricum*) from single trees at the botanic garden of Ulm University during April and August 2012. Between April and August 2012, we also collected branches from eight trees of *A. pseudoplatanus* at the same location. Sample collection for all species took place between 8 and 9 am to avoid severe water stress levels and high levels of native embolism. Although minor changes in the effect of seasonality cannot be completely excluded, differences in the ionic effect between April and September were found to be insignificant (Gascó *et al.* 2007). Moreover, pit membrane chemistry has been reported to differ between the growing and the non-growing season (Wheeler 1981; Pesacreta *et al.* 2005), but is unknown to show considerable differences between spring and summer. All trees sampled were older than ten years. Collected branches were cut in the field and transported to the lab in a plastic bag with wet tissue within ten minutes. For all experiments, branches were recut under water prior to measurements. For the anatomical measurements and immunolocalisation, we focused on the last (i.e. current year) growth ring.

### Vessel length measurements

We used the silicone injection method to assess the vessel length distribution (Sperry *et al.* 2005; Scholz *et al.* 2013b). Five branches per species were collected and trimmed to a length of 30 cm. The branches had a minimum diameter of 8 mm. Stem segments were perfused at 0.175 MPa with commercial bottled water (Auvergne Regional Park, France) at room temperature for 30 min, or until no air bubbles could be seen at the open end. We used a two-component silicone system (Rhordosil ESA 7250 A and ESA 7250 B, Bodo Müller GmbH). Both substances were mixed in an 11:1 ratio (A to B). The colourless silicone was stained by adding 1 % (w/v) Uvitex (Ciba UK plc, Bradford, UK) dissolved in chloroform. The silicon mixture was degassed for 20 min, or until no gas bubbles emerged. Silicon was injected in the stem segment with a Modell 100 pressure chamber instrument (PMS, Oregon, USA). Small amounts of the silicon mixture were poured in glass vials. The distal end of braches were submerged in the silicon mixture and transferred to a pressure chamber, which was then pressurized to 0.2 MPa for 2 h. The silicon was allowed to polymerize for 2 h at room temperature and transverse sections were made with a sliding microtome (GLS, Birmensdorf, Switzerland). Vessel length distribution was assessed by investigating these sections, starting at the proximal end. The first positive silicone observation in a vessel was considered to represent the maximum vessel length. We used the maximum vessel length to calculate four additional distances to estimate the vessel length distribution, with 6 mm as the minimal distance (Sperry *et al.* 2005).

### Hydraulic measurements of branch segments

Commercial bottled water (Auvergne Regional Park, France) was used as a reference solution for our hydraulic measurements to avoid artefacts caused by low salt concentration (Sperry *et al.* 2005; van Ieperen 2007). According to data from the supplier, this reference solution included 0.504 mM Na^+^, 0.286 mM Ca^+2^, 0.07 mM Mg^2+^, 0.158 mM K^+^, 0.084 mM ${\text{SO}}_{\text{4}}^{\text{2}}$^−^ and 1.58 mM HCO_3_^−^, while the pH was 7. Samples were perfused with this solution at 0.2 MPa for at least 30 min, or until no air bubbles emerged from the open end. This flushing was required to refill embolised conduits. As the magnitude of the ionic effect is influenced by the percentage of intact conduits in stem samples (Gascó *et al.* 2006), the stem-specific hydraulic conductivity (*K*s, kg s^−1^ m^−1^ MPa^−1^) was measured on stem segments that corresponded to 80 % of the average vessel length (ranging from 2.27 to 4.81 cm), which means that most vessels were closed and had no open vessel ends in the sample. This approach allowed us to make a direct comparison across the six *Acer* species, because this method takes into account the distribution of the vessel length classes (Gascó *et al.* 2006). We perfused the samples with a pressure of 0.007 MPa in a Sperry apparatus (Sperry *et al.* 1988). The flowrate of water was monitored each 5 s with an Sartorius CPA 225D balance. If the flow rate showed less than 5 % variation over 30 s, the flow was considered to be stable and the hydraulic conductanctivity was measured over 1 min. In most cases, stable flow rates were obtained after 15 min. We tested the ionic effect by comparing stem-specific conductivity (*K*_S_) with the reference solution and a high-salt solution, which consisted of commercial water with an additional 25 mM KCl, and calculated the ionic effect (%) as the increase in conductivity. The xylem surface area was measured after the hydraulic measurements were completed. Callipers were used to measure the xylem diameter, which allowed us to calculate the xylem surface area. The pith area could be neglected because of its small area in our stem segments.

### Immunolocalisation of cell-wall components

Fluorescence microscopy was applied using a set of six rat monoclonal antibodies: LM18 (pectic HG; Verhertbruggen *et al.* 2009), LM11 (heteroxylan; McCartney *et al.* 2005), LM2 (AGP glycan; Smallwood *et al.* 1996; Yates *et al.* 1996), JIM13 (AGP glycan; Knox *et al.* 1991), JIM20 (extensin; Smallwood *et al.* 1994; Knox *et al.* 1995) and LM1 (extensin, Smallwood *et al.* 1995). As far as we know, this is the first study applying the antibodies LM1, LM18, JIM20 and JIM13 to intervessel pit membranes. Possible masking effects restricting access to cell-wall components (e.g. Marcus *et al.* 2008) were not considered in this study.

Slivers of wood from the current growth ring were fixated overnight in a solution with 4 % paraformaldehyde, 0.1 mM phosphate buffer and 1 % sucrose at pH 7.3. Samples were embedded in LR-Gold resin following the instructions of the manufacturer. We tested cell-wall epitope distribution by fluorescence microscopy. Semi-thin sections (0.5 µm) were mounted on an object slide and incubated for 30 min in 5 % (w/v) milk protein in phosphate-buffered saline (PBS). A 5-fold dilution of the primary antibody in milk/PBS replaced the blocking milk. After an hour, we washed the sample three times with PBS. The secondary antibody goat anti-rat-IgG conjugate with fluorescein isothiocyanate (FITC) was diluted 100-fold and incubated in darkness for 1 h in milk/PBS. Unbound antibody was removed by washing samples three times with PBS for 5 min. The sections were heat-fixed to glass slides without a mounting medium. We included controls to test non-specific binding of the secondary antibody. Additionally, we tested the sample for auto-fluorescence, which was quenched by staining with toluidine blue. Samples showing no auto-fluorescence were treated with calcofluor white to enhance the contrast of cell walls.

### Wood anatomy

Wood anatomical features related to the dimensions and quantity of pits and vessels were measured following standard protocols (Scholz *et al.* 2013b;). Transverse sections with a thickness of ca. 25 µm were prepared with a sliding microtome (GLS, Birmensdorf, Switzerland). Staining of the sections was performed with a 1 % (w/v) safranin solution in 50 % ethanol and a 1 % (w/v) alcian blue solution in demineralised water. After staining, the samples were dehydrated in an ethanol series (50 %, 70 % and 96 %), treated with Neo-clear clearing agent (Merck Millipore, Germany), and embedded in Neo-mount (Merck Millipore, Germany). The embedding medium was polymerized in an oven at 60 °C overnight. Photographs of the latest growth rings were taken with a Leica DM RBE microscope system (Leica Micosystems, Wetzlar, Germany).

Electron microscopy, including scanning electron microscopy (SEM) and transmission electron microscopy (TEM), was applied to investigate ultra-structural details of pits and cell walls. Tangential sections of about 1 cm^2^ and a maximum thickness of 3 mm were oven dried overnight and mounted on SEM stubs using carbon cement. The stubs were sputtered with a thin layer of gold using a Balzers Union sputter coater (Lichtenstein, Lichtenstein). SEM pictures were obtained with a Zeiss DSM 942 SEM-system (Jena, Germany).

For TEM observations of the pit membrane thickness (*T*_PM_) and vessel wall thickness (*T*_VW_), slivers from short branch segments (5 mm) were transferred to Karnovskýs fixative at room temperature. After washing with 0.1 M phosphate buffer, samples were postfixed in 1 % buffered osmium tetroxide (OsO_4_) for 4 h at 5 °C. The OsO_4_ was removed by washing with phosphate buffer and a graded ethanol series (30 %, 50 %, 60 %, 70 %, 90 % and 96 % ethanol) was applied to dehydrate the samples. The ethanol was then gradually replaced with Epon™ resin over several days. The samples were cut with an ultramicrotome (Ultracut, Reichert-Jung, Austria) to obtain transverse, semi-thin sections of 500 nm. Ultrathin sections were observed with a Jeol JEM-1400 TEM (München, Germany).

### Statistical analysis

Anatomical characters and hydraulic measurements (*K*_s_ and ionic effect) were expressed by average values (± standard deviation) based on at least five measurements per species. The correlation between a xylem feature anatomical and the ionic effect was tested by calculating a Pearson correlation coefficient with *P* = 0.05 as a threshold value. The IBM SPSS Statistics version 20 (2011, SPSS Inc., Chicago, IL, USA) was used for the analyses.

## Results

### Hydraulic measurements of branch segments

**Figure 1.**
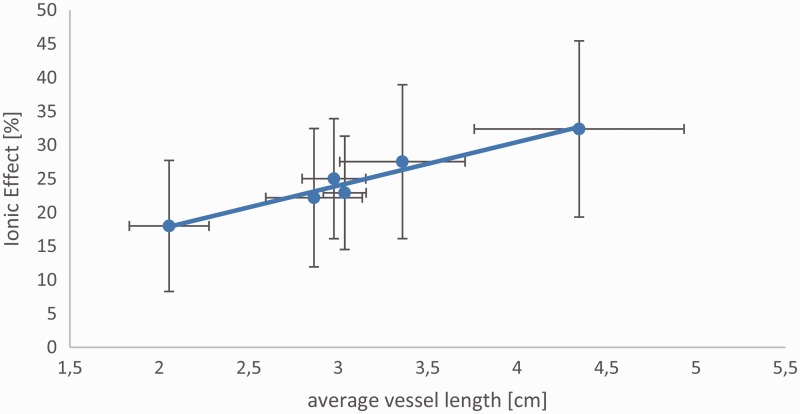
Relation between mean vessel length (LV) and mean relative increase in hydraulic conductivity (“ionic effect”, in %) of the six *Acer* species studied. Pearson correlation coefficient *r*= 0.84, *P*= 0.03).

The stem specific hydraulic conductivity (*K*_s_) using the reference solution varied from 0.269 (± 0.018) kg s^−1^ m^−1^ MPa^−1^ (mean ± SD) in *A. monspessulanum* to 0.367 (± 0.024) kg s^−1^ m^−1^ MPa^−1^ in *A. sieboldianum*. All species showed a significant increase in *K*_S_ when perfusing the samples with the 25 mM KCl solution (see **[Supporting Information —Table S1]** and Fig. 1). The ionic effect measured was on average 24.7 % (± 12.4) and ranged from 18.0 % (± 9.7) in *A. palmatum* to 32.4 % (± 13.1) in *A. tataricum* (Fig. 1).

### Immunolocalisation of cell wall polysaccharides

**Figure 2.**
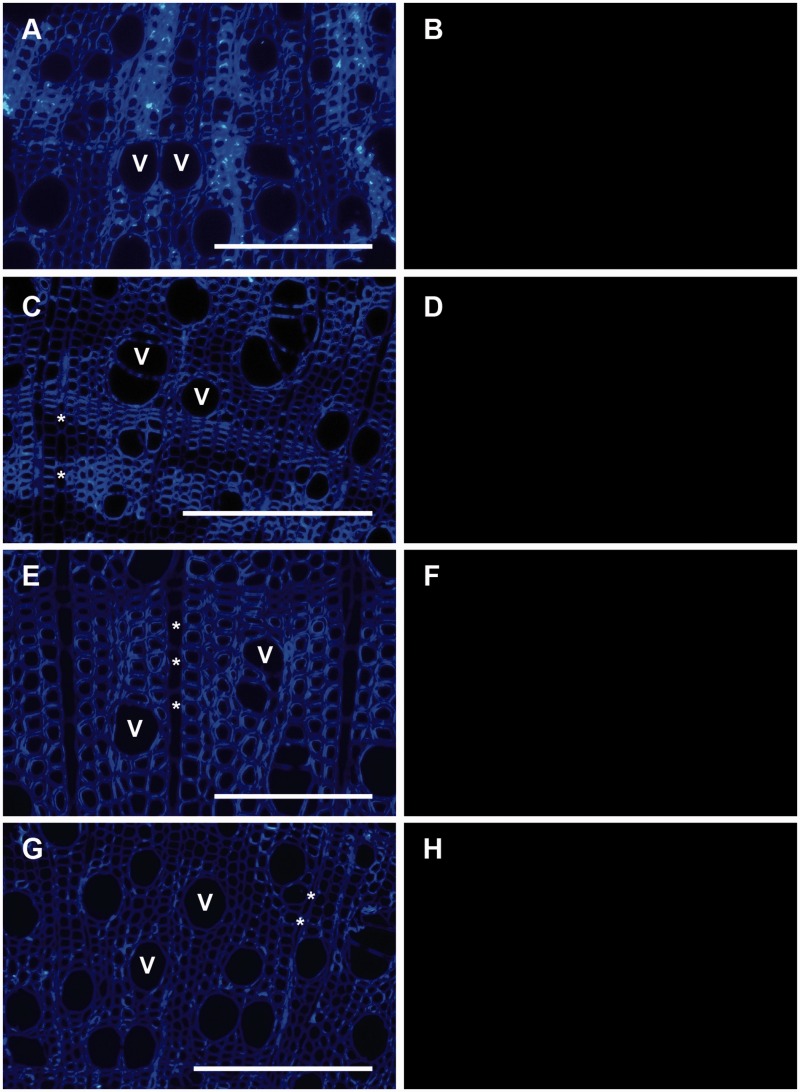
Selection of lack of epitope detection for the antibodies LM2 (AGP glycan, A and B), LM1 (extensin, C and D), JIM20 (extensin; E and F) and LM18 (HG; G and H) applied to transverse wood sections. Species include *A. tataricum* (A–C, G and H) and *A. sieboldianum* (C and D, E and F). Micrographs on the left (A, C, E and G) show the sections stained with calcofluor white, while fluorescence images are shown on the right (B, D, F and H). V, vessel; *Ray parenchyma cells. Scale bar = 100 µm.

**Figure 3.**
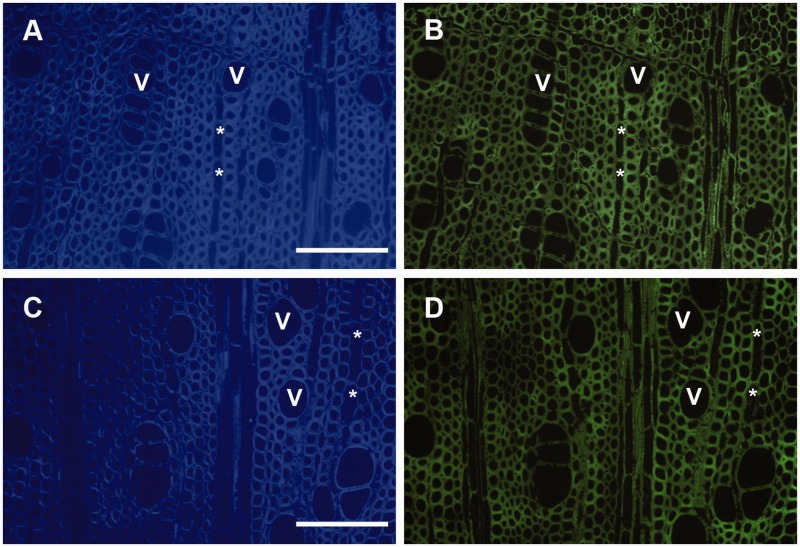
Selection of immunohistological observations with the anti-xylan antibody (LM11) in *A. monspessulanum* (A) and *A. palmatum* (C). Micrographs on the left (A and C) show the transverse wood sections stained with calcofluor white, the localisation of the antibody under fluorescent light on the right (B and D). No positive staining can be seen in the vessels (= V), ray parenchyma (= *), and intervessel walls (arrows). Scale bar = 100 µm.

**Figure 4.**
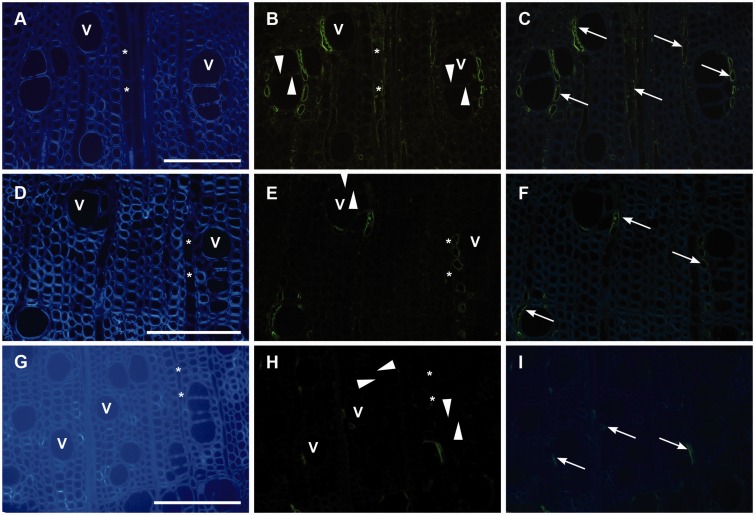
Selection of immunohistological observations with the anti-AGP antibody (JIM13) in *A. palmatum* (A and B), *A. sieboldianum* (C and D) and *A. tataricum* (E and F). Micrographs on the left (A, C and E) show the transverse wood sections stained with calcofluor white, the fluorescence images are shown on the right (B, D and F). Positive signals (arrows) for AGP can be seen in axial parenchyma cells (=*) associated with vessels (=V), ray parenchyma, but not in intervessel pit membranes (triangles) Scale bar = 100 µm.

Results from the immunolocalisation are summarized in Table 2. The controls included for non-specific binding of the secondary antibody were negative for all species studied. No positive staining could be detected for LM1 (extensin), LM2 (AGP glycan), JIM20 (extensin) and LM18 (HG; Fig. 2). From the six antibodies tested, LM11 (xylan, Fig. 3) and JIM13 (AGP, Fig. 4) showed positive staining of the xylem tissue. The antibody LM11 labelled each xylem cell wall, indicating the ubiquitous but weak distribution of xylan in their secondary cell walls.

**Table 2.** Immunolocalisation of six antibodies in secondary xylem tissue of six *acer* species. “ +++” = strong signal of the corresponding epitope; “+” = signal was detected, “± “= weak detection of the epitope, and ‘−’= no signal. *A. cam* = *A. campestre*, *A mon* = *A. monspessulanum*; *A. pla* = *A. palmatum*; *A. pse* = *A. pseudoplatanus*; *A. sie* = *A. sieboldianum*; *A. tar* = *A. tartaricum*.

Species	Control	LM2	JIM13	LM1	JIM20	LM18	LM11
*A. cam*	−	−	+	−	−	−	+++
*A. mon*	−	−	+	−	−	−	+++
*A. pla*	−	−	+++	−	−	−	+++
*A. pse*	−	−	+	−	−	−	+++
*A. sie*	−	−	±	−	−	−	+++
*A. tat*	−	−	+	−	−	−	+++

The JIM13 (AGP glycan) epitope was present in ray and axial parenchyma cells, but most pronounced in vessel-associated parenchyma cells of all six *Acer* species. Its distribution was consistently observed near the inner cell wall of vessel-associated parenchyma cells (Fig. 4). The staining intensity varied between the species studied (Table 3). In *A. palmatum*, the JIM13 epitope was clearly seen in parenchyma cells (Fig. 4A–C), while weaker signals were detected in *A. sieboldianum* (Fig. 4D–F) and *A. tataricum* (Fig. 4G–I).

**Table 3.** Wood anatomical features of six *Acer* species. All numbers represent mean values ±SD. * = no standard deviation is given for *V*_G_ and *V*_S_, which were measured on 100 individual vessels in two to three transverse wood sections. *A. Cam, a. Campestre*; *a. Mon, a. Monspessulanum*; *a. Pla, a. Platanatum*; *a. Pse*, *a. Pseudoplatanus*; *a. Sie, a*. *Sieboldianum*; *a. Tar, a. Tataricum*. Character acronyms follow **[Supplementary Table 2].**

Character (units)	*A. cam*	*A. mon*	*A. pla*	*A. pse*	*A. sie*	*A. tat*
*A*_P_ (mm^2^)	0.60 ± 0.24	0.36 ± 0.12	0.37 ± 0.12	0.61 ± 0.24	0.63 ± 0.18	0.73 ± 0.2
*A*_PF_	2.06 ± 0.45	1.75 ± 0.40	1.66 ± 0.35	1.68 ± 0.28	2.03 ± 0.47	1.55 ± 0.42
*A*_Pit_ (µm^2^)	23.29 ± 5.04	25.23 ± 3.61	18.54 ± 2.70	34.18 ± 6.62	19.15 ± 3.24	21.23 ± 3.37
*A*_Pit Ap_ (µm^2^)	1.99 ± 0.37	1.34 ± 0.35	1.24 ± 0.32	2.95 ± 0.93	1.62 ± 0.34	0.97 ± 0.27
*A*_V_ (mm^2^)	3.10 ± 0.83	2.01 ± 0.53	1.97 ± 0.52	2.84 ± 0.81	2.84 ± 0.72	3.40 ± 0.77
*D* (µm)	23.39 ± 5.68	21.07 ± 5.39	27.65 ± 6.45	41.9 ± 11.16	29.86 ± 7.44	22.48 ± 5.03
*D*_H_ (µm)	28.28 ± 6.86	26.23 ± 6.81	33.14 ± 7.73	42.32 ± 11.16	34.56 ± 8.61	27.14 ± 6.08
*F*_C_	0.28 ± 0.05	0.28 ± 0.05	0.29 ± 0.04	0.31 ± 0.08	0.32 ± 0.04	0.30 ± 0.05
*F*_LC_	0.18 ± 0.03	0.32 ± 0.03	0.37 ± 0.06	0.57 ± 0.08	0.42 ± 0.02	0.20 ± 0.01
*F*_P_	0.19 ± 0.04	0.18 ± 0.03	0.19 ± 0.03	0.21 ± 0.03	0.22 ± 0.03	0.22 ± 0.03
*F*_PF_	0.69 ± 0.05	0.65 ± 0.01	0.66 ± 0.05	0.69 ± 0.06	0.69 ± 0.04	0.71 ± 0.02
*L*_C_ (cm)	0.60 ± 0.07	0.94 ± 0.06	0.77 ± 0.09	1.63 ± 0.17	1.28 ± 0.06	0.96 ± 0.04
*L*_V_ (cm)	4.21 ± 0.46	3.04 ± 0.20	2.27 ± 0.13	2.86 ± 0.30	3.12 ± 0.05	4.81 ± 0.18
*T*_PM_ (nm)	188 ± 37	225 ± 29	201 ± 40	235 ± 26	192 ± 11	146 ± 16
*T*_VW_ (µm)	2.63 ± 0.59	3.52 ± 0.82	2.48 ± 0.48	6.427 ± 1.07	2.17 ± 0.51	1.97 ± 0.49
*V*_D_ (mm^2^)	125 ± 27	120 ± 40	104 ± 28	83 ± 13	187 ± 11	239 ± 27
*V*_G_ *	1.33	1.54	1.64	1.52	1.84	1.27
*V*_S_ *	0.82	0.68	0.63	0.43	0.58	0.80

### Anatomical observations

A survey of the anatomical characters of the xylem cells is provided in Table 3. The mean vessel diameter (*D*) was consistent for most species and around 25 µm, except for *A. pseudoplatanus*, which showed a more variable and wider diameter of 42 (± 11) µm. The latter species also showed the lowest vessel density (*V*_D_) with 82 (±13) vessels per mm^2^, and the thickest intervessel walls (*T*_VW_), which were 6.4 (± 1.1) µm. *Acer campestre*, *A. monspessulanum* and *A. palmatum* showed vessel density values (*V*_D_) around 120 vessels per mm^2^, while *A. sieboldianum* and *A. tataricum* had more than 180 vessels per mm^2^.

Differences in the solitary vessel index (*V*_S_) ranged from 0.82 for *A. campestre*, which means that 82 % of all vessels counted were solitary, to 0.43 in *A. pseudoplatanus*. The vessel-grouping index (*V*_G_) ranged between 1.27 in *A. tataricum* and 1.84 in *A. sieboldianum*. The average vessel length (*L*_V_*)* varied from 2.27 (± 0.13) cm in *A*. *palmatum* to 4.81(± 0.18) cm in *A. tataricum*, while *L*_V_ was around 3 cm for *A. monspessulanum*, *A*. *pseudoplatanus* and *A*. *sieboldianum*.

Little variation was found for the intervessel pit-field fraction (*F*_PF_), with values ranging from 0.65 (± 0.01) to 0.71 (± 0.02) in *A. monspessulanum* and *A. tataricum*, respectively. The average surface area of a single intervessel pit membrane (*A*_Pit_) was between 21.23 (± 3.4) µm^2^ in *A. tataricum* and 34.18 (± 6.6) µm^2^ in *A. pseudoplatanus*. The pit membrane thickness (*T*_PM_) varied considerably, with relatively thin membranes of 146 (± 16) nm in *A. tataricum* to 235 (± 25) nm in *A. pseudoplatanus.* The pit aperture area (*A*_Pit AP_) showed a constant intraspecific variation (SD = ±0.3 µm), except for *A. pseudoplatanus*, which also had the largest pit aperture area (*A*_Pit ap_) of 2.95 (± 0.93) µm^2^.

We found a strong correlation between the average ionic effect and the mean vessel length *L*_V_ (Pearson’s *r* = 0.84, *n* = 6, *P* = 0.03). No other anatomical features (*D*, *D*_H_, *V*_D_, *V*_G_, *A*_P_, *A*_Pit_, *A*_Pit ap_, *F*_PF_,*T*_PM_ and *F*_P_) correlated with the ionic effect values of the six *Acer* species.

## Discussion

### Testing the hydrogel hypothesis

One of the key findings of this paper is that none of the epitopes for the six antibodies tested could be detected in intervessel pit membranes of the six *Acer* species studied. The lack of HG and RG-I-related epitopes in intervessel pit membranes as based on LM18 and seven additional antibodies tested in previous studies (Table 1; Plavcová and Hacke 2011; Kim and Daniel 2012, 2013; Herbette *et al.* 2015; but see Sun *et al.* 2011) suggest that pectic polysaccharides appear to be absent in intervessel pit membranes of fully developed vessels, and that the hydrogel hypothesis does not fully explain the ionic effect. Therefore, an alternative hypothesis is required, which supports our scenario 2 as outlined in the Introduction, but rejects scenario 1 (Nardini *et al.* 2007b, 2011; van Doorn *et al.* 2011; Santiago *et al.* 2013). New functional explanations for the ionic effect could for instance come from surfactants and surfactant-coated nanobubbles in xylem sap, which may change in size depending on the ionic concentration of xylem sap (Duval *et al.* 2012; Jansen and Schenk 2015; Schenk *et al.* 2015). A lack of pectins and removal of the non-cellulosic, non-pectic components during vessel development was also suggested based on traditional staining techniques (O’Brien and Thimann 1967; O’Brien 1970). However, the presence of pectins in vessel-parenchyma pit membranes has been reported several times (Table 1; Plavcová and Hacke 2011; Kim and Daniel 2012; Kim and Daniel 2013; Herbette *et al.* 2015), indicating that pit membrane chemistry also depends on the pit type. While the occurrence of pectins in vessel–parenchyma pit membranes could be associated with gel and tylosis formation (Rioux *et al.* 1998; De Micco *et al.* 2016), these pectins are unknown to have any effects on the ionic effect. We could not detect pectins in the half-bordered vessel-parenchyma pits of *Acer* (via LM18).

The presence of cellulose in fully developed intervessel pit membranes was proven in functional assays and based on histological observation with specific probes for crystalline and non-crystalline cellulose (Dusotoit-Coucaud *et al.* 2014; Herbette *et al.* 2015). Few studies, however, have suggested that methyl-esterified pectins remain present in mature intervessel pit membranes of *Acer pseudoplatanus*, *Dianthus caryophyllus*, *Populus italica* and *Robinia pseudo-acacia*, while acidic pectins and vic-glycol side-groups are removed from pit membranes during hydrolysis (Catesson *et al.* 1979; Catesson 1983). The presence of acidic pectins has been reported in torus-bearing pit membranes of *Ulmus* (Czaninski 1979; Jansen *et al.* 2004). Based on the ruthenium red staining and hydroxylamine-ferric chloride staining techniques, the relative abundance of acidic versus methylesterified pectins was suggested to be closely related to the ionic effect in four Lauraceae species (Gortan *et al.* 2011). Light microscopic observation after staining with ruthenium red also suggested that pectins occur in the intervessel pit membranes of *Umbellularia californica* (Nardini *et al.* 2011). However, these more traditional staining techniques should be interpreted with caution because the classical reaction of ruthenium red with pectins is typical but not highly specific (Bonner 1946; Luft 1971).

Evidence for the lack of pectic polysaccharides in intervessel pit membranes based on immunocytochemical techniques appears to be consistent (Table 1). Application of a commercial pectinase treatment to intervessel pit membranes in *Fagus sylvatica* did not affect the pit membrane ultrastructure in TEM, and was found to have no effect on embolism resistance, unlike cellulase-treated material (Dusotoit-Coucaud *et al.* 2014). The observation that hydrolysis of pectins induced a sharp increase in vulnerability to embolism, without any significant effect on hydraulic conductance, could be caused by the occurrence of pectins in the pit membrane annulus (Plavcová and Hacke 2011; Kim and Daniel 2013). However, methylesterified HG and fucosylated xyloglucans (XyGs) were detected in intervessel pit membranes of grapevine plants based on the JIM7 and CCRC-M1 antibodies, respectively (Sun *et al.* 2011). A potential explanation for this finding of pectins in intervessel pit membranes could be that the observations by Sun *et al.* (2011) are based on juvenile xylem of young branches (< 12 weeks old), which may include a high amount (ca. 35 %) of living, not fully differentiated vessels (Jacobsen *et al.* 2015). The observation of methylesterfied HG and XyGs in grapevine should, therefore, be tested in mature xylem tissue.

Heteroylans (LM11) appear to be abundantly distributed in the secondary cell wall of all xylem cells (Awano *et al.* 2002). The presence of the JIM13 AGP epitope characterises xylem parenchyma cells, including both ray and axial parenchyma (Fig. 2), while LM2 targets a different AGP epitope that appears to be absent. AGPs have also been reported in meta- and protoxylem vessels of *Echinacea purpurea* using antibodies against (β-D-Glc)3 Yariv phenylglycoside (Göllner *et al.* 2013). AGPs perform various functions in plants: they are involved in growth, programmed cell death, pattern formation, and interact with growth regulators (Seifert and Roberts 2007). In some parenchyma cells, we detected AGPs in the plasma membrane based on the JIM13 epitope. The appearance of AGPs in the plasma membrane is a logical consequence of glycosylphosphatidylinositol (GPI) anchoring in the plasma membrane. Although more evidence is required, AGPs could be involved in the monitoring of the hydraulic system, formation of tyloses, refilling of embolised conduits or other hydraulic processes.

### To what extent do anatomical features account for the ionic effect?

Surprisingly, we found a positive and significant correlation between the mean vessel length and the ionic effect, but not with any other vessel and bordered pit characteristics. This finding suggests that species with longer vessels such as *A. tataricum* have a stronger ionic effect than species with a shorter mean vessel length. For *Populus tremula*, *Tilia cordata* and *Acer platanoides*, a negative correlation between xylem conduit diameter and ionic effect has been reported (Aasamaa and Sõber 2010). Considering that vessel diameter and length are positively related (Hacke *et al.* 2006), these findings appear not to agree with our measurements. Hence, further research based on a larger number of species and a wide range of vessel lengths would be required to test the correlation reported here.

The lack of other anatomical correlations appears to contradict earlier work on four *Acer* species (Nardini *et al.* 2012), including three species that were also investigated in this study (i.e. *A. campestre*, *A. monspessulanum* and *A. pseudoplatanus*). A positive correlation was found between the ionic effect and characters related to vessel grouping and intervessel conectivity (Jansen *et al.* 2011; Nardini *et al.* 2012). A correlation between the ionic effect of four *Acer* species and the intervessel contact fraction (*F*_C_) was only supported at the interspecific level and not significant at the intraspecific level (Nardini *et al.* 2012). A potential explanation for this discrepancy could be that the six *Acer* species studied here show a relatively narrow range of variation in the ionic effect (from 18 % in *A. palmatum* to 31 % in *A. tataricum*) compared with the 2–32 % range across 20 species (Jansen *et al.* 2011). However, a similar narrow range from 15 % to 23 % was also reported by Nardini *et al.* (2012). Moreover, the ionic effect of the four *Acer* species measured by Nardini *et al.* (2012) were based on stem segments that were ca. 10 cm long, while our measurements were based on a stem segment length with at least 80 % of all vessels intact (i.e. closed). Therefore, direct comparison between this study and Nardini *et al.* (2012) cannot be made.

## Conclusions

This paper demonstrates the ionic effect for six, closely related species within the genus *Acer*. Although this phenomenon has various implications in the field of plant water relations, the actual relevance *in planta* and the potential relationships between the ionic effect and plant traits have not been elucidated. Our results confirm the absence of pectic polysaccharides in intervessel pit membranes and the lack of a relation with several pit anatomical traits, which reinforces the need for an alternative hypothesis besides the hydrogel hypothesis to provide a full mechanistic explanation of the ionic effect.

## Sources of Funding

Our work was founded by the Juniorprofessor-Programm of the Baden-Württemberg (Germany).

## Contributions by the Authors

M.M.K. and M.S. conducted all immunolabelling work, hydraulic measurements and anatomical observations. M.M.K. and S.J. planned the experiments and observations, and J.P.K. assisted with the immunolabelling. All authors contributed substantially to the writing.

## Conflicts of Interest Statement

The authors report that they no conflicts of interest.

## Supporting information

The following additional information is available in the online version of this article —

**Table S1**. Hydraulic conductance values for six Acer species before and after adding 25 mM KCl to a reference solution. Student’s *t*-test statistics are given for dependant samples.

**Table S2**. Anatomical features measured with their acronyms and definitions based on Scholz *et al.* (2013).
